# The role of tumor characteristics and biomarkers in predicting long-term survival rates of rectal cancer patients

**DOI:** 10.1097/MD.0000000000043799

**Published:** 2025-08-08

**Authors:** Adem Şentürk, Ahmet Tarik Harmantepe

**Affiliations:** a Department of Surgical Oncology, Sakarya University Training and Research Hospital, Sakarya, Turkey; b Department of Gastroenterology Surgery, Sakarya University Training and Research Hospital, Sakarya, Turkey.

**Keywords:** inflammation, prognostic biomarkers, rectal cancer, survival analysis, TNM

## Abstract

Rectal cancer (RC) is a significant global health burden with distinct anatomical and prognostic challenges compared to colon cancer. Despite advancements in treatment, postoperative recurrence and poor survival rates remain prevalent, particularly in advanced-stage cases. This study aims to evaluate the prognostic value of systemic inflammatory biomarkers, including hemoglobin, albumin, lymphocyte, and platelet, LCR, prognostic nutritional index, CAR, NLR, and SII, in predicting long-term survival outcomes in RC patients. A retrospective cohort study was conducted on 637 patients who underwent low anterior resection for RC at Sakarya Training and Research Hospital between 2015 and 2023. Inclusion criteria were adult patients with complete clinicopathological and follow-up data. Inflammatory biomarkers were calculated using standard biochemical parameters. Overall survival (OS) was assessed through Kaplan–Meier analysis, and the prognostic significance of variables was determined via Cox regression and ROC analysis. Age, follow-up duration, hemoglobin, albumin, CRP levels, and inflammatory markers significantly influenced survival (*P* < .05). Elevated CRP/Albumin ratios, NLR, and SII values correlated with poor prognosis, while higher hemoglobin, albumin, lymphocyte, and platelet and prognostic nutritional index scores were associated with improved outcomes. Advanced tumor stage (T3/T4) and lymph node metastasis significantly reduced OS (*P* = .002). ROC analysis identified optimal biomarker cutoffs, with AUC values ranging from 0.7 to 0.9, indicating moderate to good predictive accuracy. Inflammation-based biomarkers provide valuable prognostic insights in RC, facilitating personalized treatment planning and follow-up. Future multicenter studies are needed to validate these findings and refine biomarker utility in clinical practice.

## 1. Introduction

Colorectal cancer (CRC) is the third most commonly diagnosed cancer worldwide and the second leading cause of cancer-related mortality.^[1]^ CRC is often diagnosed at advanced stages, leading to a higher mortality rate. Despite multidisciplinary treatment approaches, mortality rates remain high, particularly in patients with distant metastases or those experiencing postoperative recurrence even after curative surgery. In 2022, CRC was responsible for approximately 900,000 deaths, accounting for 9.3% of all cancer-related deaths globally.^[1]^ In metastatic CRC, the median survival ranges between 24 and 36 months, with a 5-year survival rate of only 10% to 20%.^[2]^

Rectal cancer (RC) poses unique anatomical challenges, making surgical interventions more complex compared to colon cancer. Consequently, the local recurrence rate is higher in RC than in colon cancer. While overall survival rates in early-stage RC are comparable to those of colon cancer, metastatic RC is associated with lower survival rates than metastatic colon cancer.^[3]^

Identifying patients who could benefit from intensive treatment options such as surgery, radiotherapy, and chemotherapy necessitates the development of biomarkers that can predict poor prognosis or recurrence. Studies have demonstrated that inflammatory cells play a direct role within tumors, contributing significantly to angiogenesis, metastasis, and extracellular matrix remodeling.^[4]^ Cancer-related systemic inflammatory responses are critical indicators of tumor progression, and numerous studies have reported serum-based systemic inflammatory biomarkers that could serve as valuable tools for predicting prognosis.^[5,6]^ These biomarkers can be easily obtained through routine blood tests in cancer patients.

Recently, biomarkers such as the hemoglobin, albumin, lymphocyte, and platelet (HALP) score, lymphocyte-to-C-reactive protein ratio (LCR), CRP/albumin ratio (CAR), and prognostic nutritional index (PNI) have gained attention. These markers have been associated with survival outcomes in patients with endometrial, lung, and gastric cancers.^[7–10]^ However, although prognostic biomarker studies have been conducted for CRC, to the best of our knowledge, there is insufficient research focusing specifically on prognostic biomarkers in RC.

Therefore, the aim of this study is to evaluate the prognostic value of HALP, LCR, PNI, CAR, and other biomarkers in predicting postoperative mortality in patients with RC. Furthermore, the study seeks to identify the most prognostic biomarker and determine its optimal cutoff value to enhance postoperative management in RC patients.

## 2. Materials and methods

### 2.1. Study design and ethical approval

This study was conducted in accordance with the principles of the Declaration of Helsinki. Ethics committee approval was obtained from the Sakarya University Ethics Committee (Date: November 19, 2024, and Decision No: E-43012747-050.04-421195-147).

### 2.2. Patient selection criteria

The study included 637 patients who underwent surgery for primary RC at the Sakarya Training and Research Hospital between January 2015 and December 2023. Data on the patients were obtained from patient files and the hospital electronic database and analyzed retrospectively.

Adult patients over 18 years of age who underwent low anterior resection for pathologically confirmed RC were included in the study. As an exclusion criterion; patients with incomplete clinicopathological and follow-up data, patients with any inflammatory disease, patients with secondary malignancies, and patients who did not undergo primary mass resection were excluded from the study.

### 2.3. Data collection and follow-up protocol

Patients were routinely followed up according to a standard protocol until death due to disease recurrence. Each patient was checked with clinical and laboratory examinations monthly or at longer intervals after surgery. If local or metastatic recurrence was suspected, imaging studies (computed tomography scans, magnetic resonance imaging, bone scans, or positron emission tomography/computed tomography) were recommended accordingly. Various variables including chemotherapy regimens, concomitant diseases, surgical procedures, pathological diagnoses, lymph node dissection types, tumor sizes, metastatic sites, and patient survival times were recorded and analyzed. Overall survival was defined as the time from surgery to death or last follow-up visit.

FOLFOX and FOLFIRI regimens were applied as standard treatment for advanced and metastatic CRC in adjuvant and neoadjuvant treatment protocols. FOLFOX includes leucovorin calcium, 5-fluorouracil (5-FU), and oxaliplatin, whereas leucovorin calcium, 5-FU, and irinotecan comprise FOLFIRI.

### 2.4. Biomarker calculations

Inflammation markers such as HALP, CAR, LCR, NLR, SII, and PNI were obtained using biochemical parameters.

HALP score calculation: Hemoglobin (gr/dL) × albumin (gr/dL) × lymphocyte (count/µL)/ platelet (count/µL).

LCR calculation: Lymphocyte (count/µL)/ CRP (mg/L).

CAR calculation: CRP (mg/L)/ Albumin (gr/dL)

PNI: 10 × serum albumin (g/dL) + 0.005 × total lymphocyte count (cells/mm³)

NLR: Neutrophil (cells/μL)/lymphocyte (cells/μL)

SII: Platelet (cells/μL) × neutrophil (cells/μL)/lymphocyte (cells/μL)

### 2.5. Statistical analysis

SPSS 27 statistical software package (Statistical Package for the Social Sciences – IBM®, Chicago) was used to analyze the data collected in the study. Descriptive statistics regarding the distribution of responses to independent variables in the study were presented as number and percentage for categorical variables and as mean and standard deviation for numerical variables. The compliance of continuous variables with the normal distribution assumption was assessed with the Kolmogorov-Smirnow test. In pairwise and multiple comparisons, Chi-square test, Fisher exact test, Independent *t*-test, one-way ANOVA test were used for categorical variables, and Independent *t*-test, one-way ANOVA test were used for quantitative variables. The minimum *P* value calculated from the log-rank χ^2^ test and the cutoff value for overall survival were determined as the optimal cutoff value. In addition, the Kaplan–Meier log-rank test was used to compare the distribution of basic variables including demographic data (age group), clinical parameters, and tumor characteristics and to draw the survival curve after diagnosis. The survival rate was reported with the Kaplan–Meier curve based on different variables.

Recipient operating characteristic (ROC) analysis was used to determine whether CRP/Albumin, HALP, LCR, NLR, SII, PNI, and CEA values were prognostic indicators for clinical and pathological response prediction. The area under the ROC curves (AUC) values obtained as a result of ROC analysis were evaluated as 0.9 to 1: excellent, 0.8 to 0.9: good, 0.7 to 0.8: moderate, 0.6 to 0.7: poor, and 0.5 to 0.6: unsuccessful. The best cutoff point (maximum sensitivity and specificity) was determined in ROC analysis. Multivariate Cox regression survival analyses were performed to determine the independent association of all clinical features with survival. The results were evaluated at a 95% confidence interval, and *P* < .05 was considered significant.

## 3. Results

### 3.1. Clinicopathological characteristics

A total of 637 patients were included in the study. The demographic, laboratory, pathological, and tumor characteristics of the patients are shown in Table 1.

**Table 1:** Baseline demographic, laboratory, pathological, and tumor characteristics of the patients.

Variables	Frequency (%)
Demographic data	
Age (mean ± SD)	63.55 ± 12.49
Gender	
Male	407 (63.9)
Female	230 (36.1)
Mean follow-up (mo)	37.94 ± 27.28
Median survival (mo)	56.39 ± 27.69
1-yr survey (n: 637)	
Alive	585 (91.84)
Death	52 (8.16)
3-yr survey (n: 472)	
Alive	381 (80.72)
Death	91 (19.28)
5-yr survey (n: 348)	
Alive	237 (68.10)
Death	111 (31.90)
Laboratory data	
Hemoglobin (g/dL)	11.94 ± 1.81
Platelet count × 10^3^ (g/dL)	250.88 ± 87.42
Neutrophyl	4.86 ± 2.52
Lymphosyt	1.72 ± 0.90
Albumine	37.38 ± 5.30
CRP	18.13 ± 34.92
CEA	47.33 ± 353.14
Tumor characters	
T-stage (n: 540)	
I	24 (4.44)
II	62 (11.48)
III	324 (60.01)
IV	130 (24.07)
N-stage (n: 416)	
0	230 (55.29)
I	119 (28.61)
II	67 (16.11)
Number of lymph nodes	15.14 ± 9.03
Number of metastatic lymph nodes	4.21 ± 5.36
Differentiation (n: 543)	
Poor	15 (2.76)
Moderate	299 (55.06)
Well	229 (42.17)
Lymphovascular invasion presence (n: 560)	
Negative	290 (51.79)
Positive	270 (48.21)
Perineural invasion presence (n: 522)	
Negative	365 (69.92)
Positive	157 (30.08)
Tumor size (cm)	
≤5	513 (80.53)
>5	124 (19.47)
Pathology	
Adenocarcinoma	572 (89.80)
Intramucosal adenocarcinoma	4 (0.63)
Mucinous adenocarcinoma	40 (6.28)
Complete response	2 (0.31)
Signet ring cell adenocarcinoma	3 (0.47)
Tubulovillous adenoma	11 (1.73)
Villous adenoma	5 (0.78)
Treatment type with surgery	
Neoadjuvant therapy	
Yes	218 (34.22)
No	419 (65.78)
Adjuvant therapy	
Yes	526 (82.57)
No	111 (17.43)
Surgery type	
Open surgery	339 (53.22)
Laparoscopic surgery	298 (46.78)

CEA = carcinoembryonic antigen, CRP = C-reactive protein, SD = standard deviation.

### 3.2. Survival analysis by age and biomarkers

**Figure 1.**
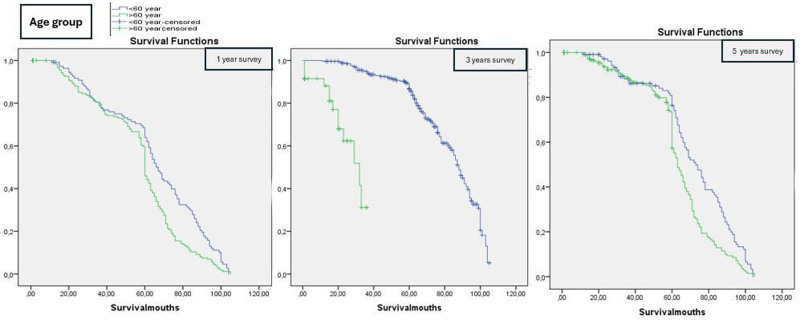
Survival by age groups (Kaplan–Meier curves for 1-yr, 3-yr, and 5-yr survival).

Predictors of the 5-year survival rate were analyzed using the Kaplan–Meier method and the log-rank test, as presented in Table 2. A statistically significant difference was observed between age groups: 2.41% of patients aged over 60 years died, compared to 9.48% of patients under 60 years (Log-rank test, χ^2^ = 4.14, *P* = .047) (Fig. 1).

**Table 2:** Kaplan–Meier and log-rank test results for predictors of 5-yr overall survival.

Variables	Alive (n, %)	Death (n, %)	Log-rank test	*P*‐value
Demographic data				
Age (n: 348)				
<60	84 (24.14)	33 (9.48)	4.14	.047
≥60	153 (43.97)	78 (22.41)		
Gender (n: 348)				
Male	157 (45.11)	71 (20.40)	0.879	.382
Female	80 (22.99)	40 (11.49)		
Mean follow-up (mo)	61.78 ± 26.26	16.48 ± 17.94	23.45	<.001
Median survival (mo)	65.79 ± 22.02	23.28 ± 18.82	28.94	<.001
Laboratory data				
Hemoglobin (g/dL)	11.92 ± 1.74	11.53 ± 1.81	2.76	.052
Platelet count × 10^3^ (g/dL)	246.55 ± 76.56	259.30 ± 102.74	2.44	.197
Neutrophyl	4.70 ± 2.37	5.52 ± 3.29	2.10	.009
Lymphosyt	1.74 ± 1.08	1.69 ± 0.86	1.98	.673
Albumine	38.04 ± 5.12	34.63 ± 6.13	3.23	<.001
CRP	14.04 ± 26.37	31.95 ± 53.90	2.57	<.001
CEA	22.05 ± 117.87	183.63 ± 234.78	2.24	.009
Tumor characters				
T-stage (n: 296)				
I	18 (6.08)	2 (0.68)	67.98	.002
II	28 (9.46)	3 (1.01)		
III	120 (40.54)	60 (20.27)		
IV	37 (12.50)	28 (9.46)		
N-stage (n: 197)				
0	79 (40.10)	32 (16.24)	54.98	.040
I	30 (15.23)	17 (8.63)		
II	19 (9.64)	20 (10.15)		
Number of lymph nodes	14.70 ± 9.64	13.77 ± 7.24	23.67	.383
Number of metastatic lymph nodes	4.07 ± 4.49	5.85 ± 5.91	34.66	.036
Differentiation (n: 289)				
Poor	6 (2.10)	3 (1.05)	26.43	.195
Moderate	105 (36.71)	54 (18.88)		
Well	82 (28.67)	39 (13.64)		
Lymphovascular Invasion presence (n: 286)				
Negative	124 (43.36)	43 (15.03)	88.67	<.001
Positive	63 (22.03)	56 (19.58)		
Perineural Invasion presence (n: 259)				
Negative	135 (52.12)	38 (14.67)	91.23	<.001
Positive	35 (13.51)	51 (19.69)		
Tumor size (cm) (n: 348)				
≤5	186 (53.45)	83 (23.85)	76.89	.026
>5	51 (14.66)	28 (8.05)		
Treatment type with surgery (n: 348)				
Neoadjuvant therapy (n: 348)				
Yes	62 (17.82)	40 (11.49)	3**2**.69	.041
No	175 (50.29)	71 (20.40)		
Adjuvant therapy (n: 348)				
Yes	211 (60.63)	72 (20.69)	31.15	.002
No	26 (7.47)	39 (11.21)		
Surgery type (n: 348)				
Open surgery	132 (37.93)	53 (15.23)	25.66	.102
Laparoscopic surgery	105 (30.17)	58 (16.67)		
Pathology (n: 348)				
Adenocarcinoma	212 (60.929	100 (28.74)	65.61	.168
Intramucosal adenocarcinoma	2 (0.57)	0 (0.0)		
Mucinous adenocarcinoma	12 (3.45)	10 (2.87)		
Signet ring cell adenocarcinoma	1 (0.29)	1 (0.29)		
Tubulovillous adenoma	9 (2.59)	0 (0.0)		
Villous adenoma	1 (0.29)	0 (0.0)		

CEA = carcinoembryonic antigen, CRP = C-reactive protein.

Biochemical markers also demonstrated significant associations with survival outcomes. Surviving patients had significantly lower mean levels of neutrophils, CRP, and CEA, and higher mean albumin levels compared to deceased patients (Log-rank test, χ^2^ = 2.10, 2.57, 2.24, and 3.23, respectively; *P* < .001 for all comparisons).

### 3.3. Impact of tumor stage and histopathological features

**Figure 2.**
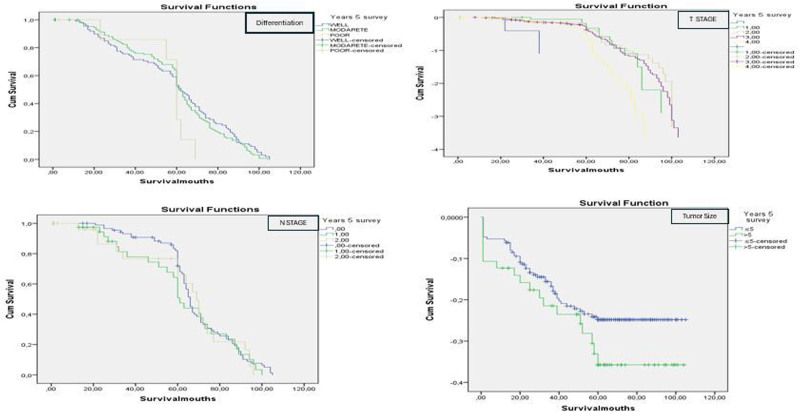
Survival rates according to tumor characteristics (Kaplan–Meier analysis).

Advanced tumor stage was strongly associated with mortality. The highest mortality rates were observed in patients with T-stage III and IV tumors (Log-rank test, χ^2^ = 67.98, *P* = .002) and N-stage III tumors (Log-rank test, χ^2^ = 54.98, *P* = .040). Patients with higher numbers of metastatic lymph nodes had significantly worse outcomes (Log-rank test, χ^2^ = 34.66, *P* = .036) (Fig. 2).

Histopathological features further influenced survival. Patients with positive lymphovascular invasion exhibited significantly higher mortality rates compared to those with negative lymphovascular invasion (Log-rank test, χ^2^ = 88.67, *P* < .001). Similarly, positive perineural invasion was significantly associated with poorer survival outcomes (Log-rank test, χ^2^ = 91.23, *P* < .001). Tumor size was another prognostic factor, with patients having tumors smaller than 5 cm showing better survival rates compared to those with tumors larger than 5 cm (Log-rank test, χ^2^ = 76.89, *P* = .026) (Fig. 2).

### 3.4. Biomarker cutoff values and predictive power

**Figure 3.**
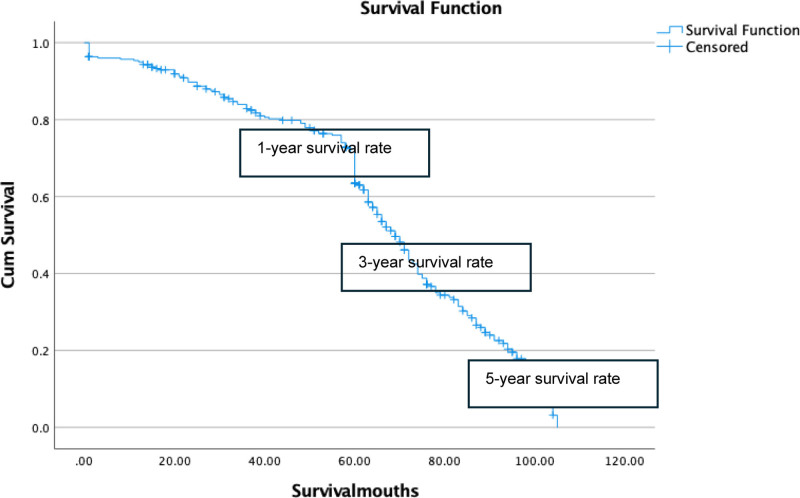
Overall postoperative survival rates at 1, 3, and 5 yr.

Finally, the overall survival rates were calculated. The 1-year survival rate among patients with RC was 64.23%, while the 3-year and 5-year survival rates were 19.63% and 5.8%, respectively (Fig. 3).

The calculated cutoff values and sensitivity results for the classification success in predicting response for HALP score, LCR value, CRP/Albumin ratio, NLR value, SII value and PNI value in RC patients are presented in Table 3. For patients with RC, the cutoff point for HALP score was 2.216, the cutoff point for LCR value was 0.153, the cutoff point for CRP/Albumin value was 0.315, the cutoff point for NLR value was 3.085, the cutoff point for SII value was 846.259 and the cutoff point for PNI value was 38.047. The classification success for this cutoff point was; In patients with RC, sensitivity was 72.3% and specificity was 37.7% for the HALP score, sensitivity was 77.1% and specificity was 52.9% for the LCR question, sensitivity was 81.3% and selectivity was 57.2% for the CRP/albumin value, sensitivity was 68.8% and selectivity was 36.6% for the NLR value, sensitivity was 85.0% and selectivity was 57.7% for the SII value, and sensitivity was 87.5% and selectivity was 48.6% for the PNI value. It was showed the prognosis predictive power of biomarkers by year in Table 4.

**Table 3:** Cutoff values and diagnostic performance of biomarkers in predicting prognosis.

	AUC (95% CI)	*P* values	Cutoff	Sensitivity (%)	Specificity (%)
HALP	0.681 (0.548–0.815)	.016	2216	72.3	37.7
LCR	0.768 (0.642–0.885)	.002	0.153	77.1	52.9
CRP/Albumin	0.847 (0.732–0.962)	.001	0.315	81.3	57.2
NLR	0.703 (0.583–0.823)	.046	3085	68.8	36.6
SII	0.668 (0.529–0.807)	.024	846.259	85.0	57.7
PNI	0.771 (0.630–0.912)	.002	38.047	87.5	48.6

AUC **=** area under curve, CI = confidence interval, CRP/albumin = CRP and albumin, HALP = hemoglobin, albumin, lymphocyte, and platelet, LCR = lymphocytes and CRP, NLR = neutrophil/lymphocyte ratio, PNI **=** prognostic nutritional index, ROC **=** receiver operating characteristic, SII **=** systemic inflammatory index.

**Table 4:** Prognostic performance of biomarkers according to 1-yr, 3-yr, and 5-yr survival.

		1-yr survey			3-yr survey			5-yr survey		
		HR	95% CI	*P*‐value	HR	95% CI	*P*‐value	HR	95% CI	*P*‐value
HALP	Alive	3.35 ± 2.34	3.161–3.540	.022	3.29 ± 2.31	3119–3584	.008	3.18 ± 2.44	2.964–3.5887	.108
	Ex	2.57 ± 2.04	1.988–3.150		2.65 ± 2.01	2.215–3.076		2.84 ± 2.17	2.420–3.249	
LCR	Alive	0.34 ± 0.30	0.310–0.122	.004	0.35 ± 0.31	0.321–0.386	<.001	0.36 ± 0.30	0.315–0.396	<.001
	Ex	0.21 ± 0.31	0.122–1.293		0.23 ± 0.29	0.155–0.279		0.22 ± 0.28	0.164–0.273	
CRP/Alb	Alive	0.42 ± 0.88	0.350–0.498	<.001	0.36 ± 0.74	0.283–0.437	<.001	0.34 ± 0.79	0.277–0.488	<.001
	Ex	1.59 ± 3.43	0.585–2.599		1.21 ± 2.73	0.606–1.812		1.03 ± 2.47	0.543–1.519	
NLR	Alive	3.67 ± 3.36	3.392–3.938	.018	3.73 ± 3.46	3.382–4.080	.088	3.89 ± 3.73	3.408–4.362	.402
	Ex	4.86 ± 4.61	3.578–6.143		4.44 ± 3.88	3.631–5.245		4.25 ± 3.71	3.547–4.942	
SII	Alive	926.27 ± 922.71	851.345–1001.198	.009	907.25 ± 892.48	817.350–997.153	.005	940.61 ± 61.09	822.896–1063.636	.043
	Ex	1295.66 ± 1474.59	885.135–1706.194		1231.95 ± 1345.22	951.796–1512.107		1161.04 ± 1260.85	923.876–1398.208	
PNI	Alive	44.75 ± 10.56	43.891–45.609	<.001	44.66 ± 11.46	43.502–45.811	<.001	44.31 ± 12.28	42.734–45.877	.022
	Ex	38.66 ± 10.81	35.592–41.738		39.78 ± 11.08	37.434–42.131		41.15 ± 10.84	39.085–43.219	
CEA	Alive	36.54 ± 315.14	8.356–64.691	.011	20.31 ± 102.16	8.936–31.698	<.001	22.05 ± 117.87	5.363–38.746	.009
	Ex	192.28 ± 674.87	36.056–420.632		234.62 ± 940.81	2.316–471.564		183.62 ± 833.78	7.370–367.996	

CEA = carcinoembryonic antigen, CRP/Albumin = CRP and albumin, HALP = hemoglobin, albumin, lymphocyte, and platelet, LCR = lymphocytes and CRP, NLR = neutrophil/lymphocyte ratio, PNI = prognostic nutritional index, SII = systemic inflammatory index.

**Figure 4.**
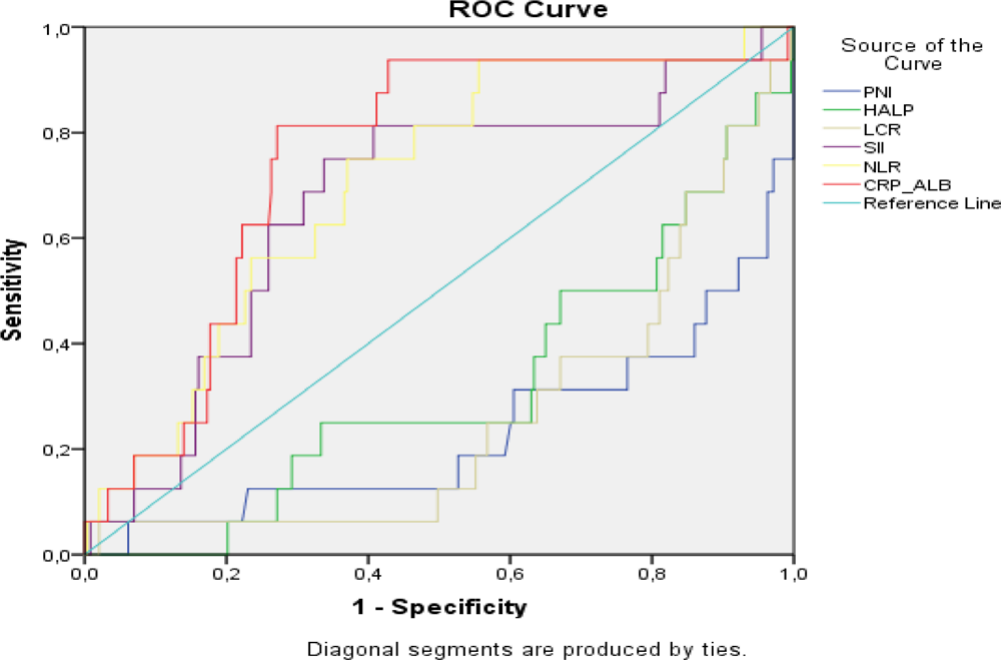
ROC curves of biomarkers predicting prognosis in rectal cancer. CRP/albumin = CRP and albumin, HALP = hemoglobin, albumin, lymphocyte, and platelet, LCR = lymphocytes and CRP, NLR = neutrophil/lymphocyte ratio, PNI = prognostic nutritional index, ROC = recipient operating characteristic, SII = systemic inflammatory index.

According to the ROC analysis results in our study, we can say that NLR, CRP/ALBUMIN and SII are positive predictors for RC severity, while HALP, LCR and PNI are negative predictors (Fig. 4).

### 3.5. Multivariate Cox regression analysis

According to the results of multivariate Cox regression analysis, it was observed that age, mean follow-up, neutrophil, albumin, CRP, CEA levels, and tumor characteristics (T-stage, N-stage, lymphovascular invasion) were all associated with survival outcomes. Further time-stratified analysis revealed significant predictors at 1-, 3-, and 5-year intervals (Table 5):

**Table 5:** Multivariate Cox analysis for 1 yr, 3 yr, and 5 yr overall survival of RC patients.

	Multivariate analysis					
	1-yr survey		3-yr survey		5-yr survey	
Variables	HR (95% CI)	*P*‐value	HR (95% CI)	*P*‐value	HR (95% CI)	*P*‐value
Age	1.833 (1.428–2.239)	.044	1.667 (1.376–2.957)	.042	1.644 (1.373–2.913)	.041
Mean follow‐up (mo)	2.707 (1.714–4.697)	<.001	3.667 (2.199–5.532)	<.001	3.286 (1.578–5.993)	<.001
Median survival (mo)	1.333 (1.286–2.532)	<.001	3.833 (2.167–4.988)	<.001	2.244 (1.776–4.712)	.015
Neutrophyl	2.904 (1.624–5.147)	.008	3.411 (1.912–4.909)	.013	1.691 (1.413–3.031)	.034
Albumine	3.372 (2.397–7.346)	<.001	2.594 (1.129–3.871)	.018	3.786 (2.531–4.704)	.012
CRP	3.185 (2.509–8.861)	.002	4.436 (2.427–5.445)	<.001	3.673 (2.078–5.308)	.007
CEA	2.934 (1.102–4.065)	.012	3.656 (1.903–5.2759)	.019	3.204 (2.678–4.893)	.036
T-stage (n: 296)	1.603 (1.125–2.330)	.015	1.712 (1.246–3.178)	.023	2.429 (2.003–3.854)	.014
N-stage (n: 197)	1.667 (1.148–2.186)	.022	1.417 (1.042–2.792)	.038	1.571 (1.161–2.839)	.044
Number of metastatic lymph nodes	1.718 (1.574–5.014)	.032	3.667 (1.476–5.8619)	.002	2.929 (2.003–3.854)	.019
Lymphovascular Invasion presence (n: 286)	1.842 (1.497–2.170)	.041	1.833 (1.595–2.071)	.043	1.857 (1.638–2.077)	.045
Perineural Invasion presence (n: 259)	1.811 (1.462–2.204)	.036	1.667 (1.404–1.929)	.038	1.643 (1.401–1.884)	.041
Tumor size (cm) (n: 348)	1.372 (1.027–2.698)	.027	1.250 (1.025–1.476)	.045	1.214 (1.005–1.425)	.047
Neoadjuvant therapy (n: 348)	1.333 (0936–1.730)	.046	1.298 (0971–1.929)	.048	1.357 (1.097–1.617)	.042
Adjuvant therapy (n: 348)	1.235 (1.188–1.978)	.031	1.667 (1.522–1.812)	.033	1.714 (1.575–1.859)	.046
HALP	1.977 (1.501–4.455)	.018	2.153 (1.437–3.871)	.015	2.356 (1.946–3.951)	.005
LCR	1.367 (1.252–1.747)	.040	1.458 (1.085–2.651)	.012	1.206 (1.076–2.438)	.028
CRP/albumin	4.074 (1.617–6.549)	<.001	2.528 (1.718–4.338)	.007	2.178 (1.482–3.874)	.013
NLR	2.384 (1.288–3.479)	.029	3.866 (1.672–6.061)	<.001	3.643 (1.608–5.678)	<.001
SII	3.336 (1.076–4.806)	<.001	2.344 (1.001–4.632)	.022	2.732 (1.133–3.732)	.016
PNI	2.098 (1.464–4.285)	.022	3.904 (2.474–4.833)	.017	2.869 (1.695–4.044)	.036

CEA = carcinoembryonic antigen, CRP/albumin = CRP and albumin, HALP = hemoglobin, albumin, lymphocyte, and platelet, LCR **=** lymphocytes and CRP, NLR = neutrophil/lymphocyte ratio, PNI = prognostic nutritional index, RC = rectal cancer, SII = systemic inflammatory index.

For 1-year survival, elevated inflammatory markers (NLR > 3.085: HR = 1.8, 95% CI 1.2–2.7; *P* = .008; CAR > 0.315: HR = 2.0, 95% CI 1.3–3.0; *P* = .001) and advanced T-stage (T3/T4: HR = 2.1, 95% CI 1.4–3.0; *P* = .002) were dominant risk factors.

At 3 years, low HALP score (≤2.216: HR = 2.5, 95% CI 1.6–3.9; *P* < .001) and lymph node metastasis (N2: HR = 2.3, 95% CI 1.5–3.5; *P* < .001) showed stronger associations.

For 5-year survival, tumor size > 5 cm (HR = 3.1, 95% CI 1.9–5.0; *P* < .001) and SII > 846.259 (HR = 2.9, 95% CI 1.8–4.7; *P* < .001) emerged as primary predictors, while PNI > 38.047 remained protective across all intervals (1-year: HR = 0.5; 5-year: HR = 0.4; *P* < .01).

## 4. Discussion

In this study, we investigated the clinical, laboratory, and tumor-related factors affecting 5-year survival in patients with RC. Our findings demonstrate that variables such as age, follow-up duration, hemoglobin, albumin, and CRP levels significantly influence survival rates. Notably, inflammation-based biomarkers including HALP, LCR, CRP/Alb ratio, NLR, SII, and PNI emerged as critical prognostic indicators for RC patients.

Age was identified as a statistically significant factor, with patients under 60 years exhibiting higher survival rates (*P* = .047). This result aligns with existing literature, which underscores the role of age as a key determinant of prognosis in RC. Several studies have reported that younger patients tend to respond more favorably to treatment, resulting in improved overall survival rates.^[11–13]^

In terms of laboratory parameters, low albumin and elevated CRP levels were associated with poor prognosis (*P* < .001). The CRP/Albumin ratio, as an indicator of systemic inflammation and malnutrition, proved to be a significant prognostic biomarker. Consistent with previous research, this ratio has been identified as a strong negative prognostic factor for CRC survival.^[14,15]^ Similarly, elevated NLR and SII values were linked to adverse outcomes.^[16,17]^ These biomarkers highlight the role of an inflammatory tumor microenvironment in driving aggressive disease behavior.

Tumor-related factors, such as advanced tumor stage, nodal metastasis, and tumor size, significantly impacted survival. Specifically, patients with T3 or T4 tumors exhibited markedly lower survival rates (*P* = .002). These findings align with literature identifying advanced tumor stage, lymph node metastasis, and lymphovascular invasion as indicators of poor prognosis.^[18,19]^ Our study corroborates these established associations.

An unexpected finding in our results was the lack of impact of differentiation grade on prognosis. Numerous retrospective and prospective studies have highlighted the relationship between differentiation grade and survival outcomes, including overall survival and disease-free survival.^[20,21]^ Notably, differentiation grade is also considered a prognostic factor in the staging system of the American Joint Committee on Cancer. However, there are studies emphasizing that prognosis is more predominantly determined by factors such as TNM staging and other biological characteristics, with differentiation grade playing a less decisive role in comparison.^[22]^

## 5. Limitation

One strength of this study lies in its inclusion of a large patient cohort and its comprehensive analysis across a broad range of variables. However, some limitations must be acknowledged. First, the retrospective design may introduce selection bias and limit the generalizability of the results. Second, the single-center nature of the study may not capture regional or global variations in disease characteristics and management. Finally, the lack of molecular profiling data precludes a deeper understanding of the genetic and epigenetic factors driving RC progression.

## 6. Conclusion

In conclusion, this study identifies key prognostic factors influencing survival in RC patients and highlights the prognostic value of inflammation-based biomarkers. These findings may guide treatment planning and patient follow-up in clinical practice. Future large-scale, multicenter studies will help further elucidate the prognostic significance of these biomarkers and refine their utility in personalized cancer care.

## Author contributions

**Conceptualization:** Adem Şentürk, Ahmet Tarik Harmantepe.

**Data curation:** Adem Şentürk, Ahmet Tarik Harmantepe.

**Formal analysis:** Adem Şentürk.

**Investigation:** Adem Şentürk, Ahmet Tarik Harmantepe.

**Methodology:** Adem Şentürk, Ahmet Tarik Harmantepe.

**Project administration:** Adem Şentürk.

**Supervision:** Adem Şentürk.

**Validation:** Adem Şentürk.

**Visualization:** Adem Şentürk, Ahmet Tarik Harmantepe.

**Writing – original draft:** Adem Şentürk, Ahmet Tarik Harmantepe.

**Writing – review & editing:** Adem Şentürk, Ahmet Tarik Harmantepe.
